# Signal Amplification-Based Biosensors and Application in RNA Tumor Markers

**DOI:** 10.3390/s23094237

**Published:** 2023-04-24

**Authors:** Haiping Li, Zhikun Zhang, Lu Gan, Dianfa Fan, Xinjun Sun, Zhangbo Qian, Xiyu Liu, Yong Huang

**Affiliations:** 1 State Key Laboratory of Targeting Oncology, National Center for International Research of Bio-Targeting Theranostics, Guangxi Key Laboratory of Bio-Targeting Theranostics, Collaborative Innovation Center for Targeting Tumor Diagnosis and Therapy, Guangxi Medical University, Nanning 530021, China; lihaiping0525@163.com (H.L.); z_z_kzhangzhikun@163.com (Z.Z.); g_lganlu@163.com (L.G.); fandf1996@163.com (D.F.); m17854337107@163.com (X.S.); qianzhangb@163.com (Z.Q.); liuxiyu419@163.com (X.L.); 2 School of Pharmacy, Guangxi Medical University, Nanning 530021, China

**Keywords:** RNA tumor markers, electrochemical biosensor, photoelectrochemical biosensor, fluorescent biosensor, signal amplification

## Abstract

Tumor markers are important substances for assessing cancer development. In recent years, RNA tumor markers have attracted significant attention, and studies have shown that their abnormal expression of post-transcriptional regulatory genes is associated with tumor progression. Therefore, RNA tumor markers are considered as potential targets in clinical diagnosis and prognosis. Many studies show that biosensors have good application prospects in the field of medical diagnosis. The application of biosensors in RNA tumor markers is developing rapidly. These sensors have the advantages of high sensitivity, excellent selectivity, and convenience. However, the detection abundance of RNA tumor markers is low. In order to improve the detection sensitivity, researchers have developed a variety of signal amplification strategies to enhance the detection signal. In this review, after a brief introduction of the sensing principles and designs of different biosensing platforms, we will summarize the latest research progress of electrochemical, photoelectrochemical, and fluorescent biosensors based on signal amplification strategies for detecting RNA tumor markers. This review provides a high sensitivity and good selectivity sensing platform for early-stage cancer research. It provides a new idea for the development of accurate, sensitive, and convenient biological analysis in the future, which can be used for the early diagnosis and monitoring of cancer and contribute to the reduction in the mortality rate.

## 1. Introduction

Today, cancer is not only one of the most prevalent diseases worldwide, but also one of the leading causes of human death. There are more than 200 types of cancer that affect the normal function of multiple human organs [1]. The rapid growth and metastasis of tumor cells pose significant problems for human beings, so early diagnosis of cancer is very important. Biomarkers are substances that can be detected in serum or tissues and are used for cancer diagnosis, post-operative monitoring, disease progression, and disease recurrence [2]. Tumor markers are substances associated with or produced by tumors that can be used to diagnose malignant tumors at an early stage. In clinical diagnosis, an elevated tumor marker concentration is an early indicator for monitoring tumor progression and detecting recurrence or metastasis. Many biomarkers associated with cancer have been found and can be used for cancer diagnosis, such as carcinoembryonic antigen (CEA) [3,4,5,6,7], alpha-fetoprotein (AFP) [8,9], prostate-specific antigen (PSA) [10], carbohydrate antigen (CA) [11,12], neuron-specific enolase (NSE) [13], etc.

With the development of bioinformatics and analysis technology, people not only pay attention to these classic tumor markers but also focus on other potential tumor markers in cells and serum, hoping to improve the accuracy of clinical tumor diagnosis. In recent years, RNA molecule tumor markers have attracted people’s interest, such as microRNA (miRNA), circular RNA (circRNA), methylated RNA (m6A-RNA), etc. MicroRNA is an endogenous non-coding RNA with a length of less than 40nt and single strand, which is not involved in protein coding. It exists in almost all human cells and plays an important role in biological processes, cell proliferation and differentiation, and carcinogenesis [14,15]. Numerous studies have shown that abnormal miRNA expression is closely associated with changes in human immune system function, leading to the germination of many malignant health-threatening diseases. CircRNA is a kind of endogenous non-coding RNA (NcRNA) with covalently closed ring structure formed by trans-splicing through the covalent 5′ end and 3′ end covalent bond produced by a special reverse splicing process [16,17,18]. Studies have shown that the expression of some circRNAs is significantly different in normal cells and tumor cells, which could make circRNAs a promising biomarker for cancer diagnosis and prognosis [19,20,21,22]. N6-methyladenosine (M6A) is one of the most widely studied and abundant RNA modifications among eukaryotic messenger RNAs [23]. A large number of studies have shown that RNA methylation plays a crucial role in post-transcriptional regulation, such as transcription splicing [24], protein translation [25], cell proliferation and differentiation [26], etc. Differences in m6A levels have certain effects on the biological functions of cells and tissues and may be related to human diseases or cancers [27]. With the rapid development of emerging technologies, especially the disadvantages of low specificity and sensitivity of traditional protein biomarkers, people have gradually realized the good substitute potential of RNA molecular tumor markers in the early screening of malignant diseases.

Analytical methods for detecting these RNA molecular markers have been developed, including northern blotting analysis [28], real-time quantitative polymerase chain reaction (RT-qPCR) detection [29,30,31], liquid chromatography-tandem mass spectrometry (LC-MS/MS) [32], m6A individual nucleotide resolved crosslinking and immunoprecipitation (miCLIP) [33], and m6A-RNA immunoprecipitation sequencing (ChIP-Seq, MERIP-Seq) [34,35]. These analytical methods provide effective ideas for the detection of RNA molecular markers, but there are still many shortcomings. The Northern blotting analysis may cause the RNA samples to be degraded by enzymes, which may affect the detection results, and is less sensitive and time-consuming compared with other techniques. The level of selectivity and specificity ability of RT-qPCR for miRNA detection needs to be improved. RT-qPCR is commonly used to verify circRNAs that have been identified by RNA sequencing and to achieve sensitive detection of circRNAs [36]. However, the use of exonuclease may lead to incomplete digestion of linear RNA and even affect the reverse transcription process, which brings trouble to the accurate detection of circRNA. Technologies such as LC-MS/MS require either expensive or large equipment or high operating costs, complex procedures, and skilled personnel, which limit the speed and accuracy of detection.

**Figure 1:**
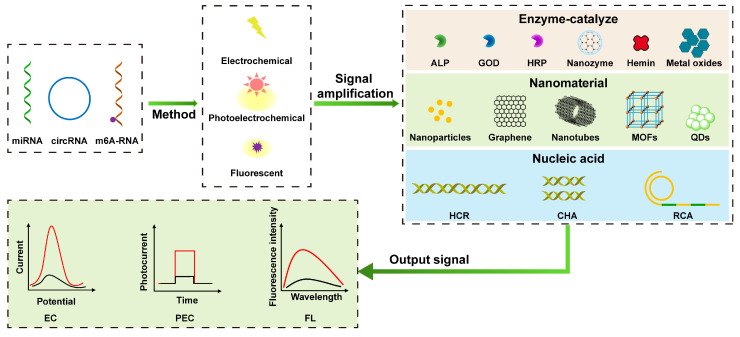
Schematic illustration of electrochemical, photoelectrochemical, and fluorescent sensing analyses based on signal amplification strategies for detecting RNA tumor markers. (The two curves represent the trend of the response signal based on signal amplification, including positive and negative correlation of signal magnification).

In recent years, biosensors have attracted a lot of interest for their superior analytical properties. Biosensors have the advantages of simple operation, high sensitivity, good selectivity, and fast response, which are beneficial for the detection of low concentrations of tumor markers in biological samples, which can help diagnose cancer at an early stage. Biosensors are mainly composed of receptors, physicochemical transducers, and signal processors [37,38]. Based on transducers, biosensors can be divided into electrochemical biosensors [39], optical biosensors [40], thermal sensitive biosensors, etc. With the continuous improvement of detection requirements, especially the interference of low detection molecular concentration and a complex detection environment, it is still a huge challenge to achieve trace or ultra-trace analysis. Therefore, various signal amplification strategies have emerged successively and been widely used to enhance detection signals, such as signal amplification based on nanomaterials, enzyme catalysis, and nucleic acid. As shown in Figure 1, after a brief introduction to different design principles of electrochemical (EC), photoelectrochemical (PEC), and fluorescent (FL) biosensors, this paper will review signal amplification strategies based on nanomaterials, enzyme catalysis, and nucleic acid. This paper reviews the latest progress of EC, PEC, and FL biosensors based on signal amplification strategies for the sensitive detection of RNA tumor markers, which provides a new idea for the early diagnosis of cancer.

## 2. Electrochemical Biosensor

Electrochemical biosensor is composed of a recognition element and a transduction element, and the detected signal is transduced into an electrical signal. The electrode system quantifies the target by evaluating the charge change caused by the binding of the analyte to the electrode surface or the current or potential response caused by the redox reaction occurring on the electrode surface. The signal amplification strategies applied to electrochemical biosensors are divided into: signal amplification based on enzyme catalysis, nanomaterials, and nucleic acid [41,42].

### 2.1. Electrochemical Sensing Analysis

**Figure 2:**
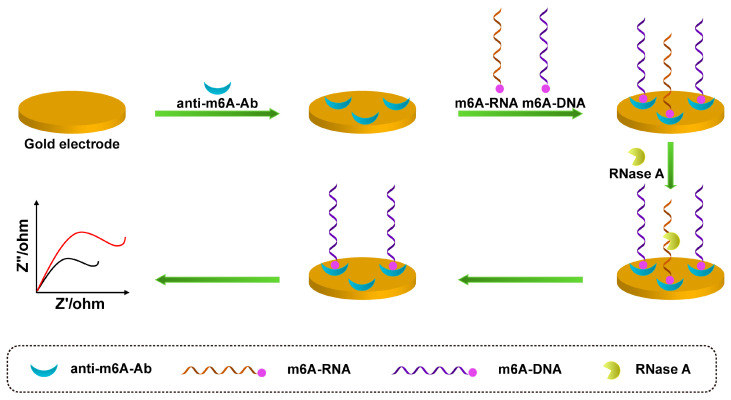
Schematic illustration of m6A-RNA competing with m6A-DNA to bind antibodies and RNase A hydrolyzing m6A-RNA for EIS detection (The red line represents the original sensing signal, the black line represents the sensing signal based on signal amplification. This is a negatively correlated signal amplification).

The electrochemical biosensor is a device that performs metrology based on the electrochemical properties of electrolyte solutions and the law of charge change of substances on the surface of electrodes. Based on the metrological relationship between electrical quantities, such as current, potential, conductance, and certain quantities of the substance to be measured, an analytical method is used to quantify different components, and this detection method is called electroanalytical chemistry. Electrochemical analysis method has high accuracy, high sensitivity, and good selectivity. In the field of electrochemical sensors, commonly used electrochemical analysis methods mainly include voltammetry, electrochemical impedance spectroscopy (EIS), the time–current curve method, etc. Voltammetry is a commonly used electrochemical method. Voltammetry is a commonly used electrochemical method. Tran et al. developed a label-free electrochemical biosensor based on a conductive polymer/reduced graphene oxide modified electrode, using square wave voltammetry (SWV) to detect redox signals for highly specific and sensitive detection of miRNA-29b-1, with detection limits of 5 fM, respectively [43]. EIS is also a commonly used method for the detection of small molecules. As shown in Figure 2, Xie et al. constructed a novel electrochemical immunosensor for detecting m6A [44]. First, according to the fact that anti-m6A-antibodies (anti-m6A-Ab) can recognize m6A-RNA and m6A-DNA, the m6A-DNA probe was used as a signal molecule to compete with m6A-RNA for antibody binding, then ribonuclease A (RNase A) hydrolyzes the RNA that bound to the antibody, and, finally, the EIS signal is detected after electrode hydrolysis. The signal intensity is inversely proportional to the amount of m6A-RNA in the sample; the detection limit was 0.016 nM.

### 2.2. Signal Amplification Strategies Used in EC Sensing

Due to the increasingly strict requirements for trace detection of target analytes in some clinical analyses and disease diagnosis, it is urgent to improve the sensitivity of biosensors. Based on the advantages of electrochemical sensors, various signal amplification strategies are used to improve the performance of electrochemical biosensors and enhance the electrochemical output signals, thus increasing the sensitivity of electrochemical biosensors.

#### 2.2.1. Enzyme-Catalyze Signal Amplification

Enzyme-catalyzed signal amplification is one of the most commonly used techniques in signal amplification strategy, which can be attributed to the advantages of enzyme-catalyzed reactions, such as high catalytic efficiency, strong specificity, and mild reaction conditions. In this technology, the enzyme is attached to the electrode surface, and the catalytic activity of the enzyme is used to catalyze the corresponding enzyme substrate to produce signal molecules to achieve signal amplification. Natural enzymes commonly used in electrochemistry include alkaline phosphatase (ALP), horseradish peroxidase (HRP), and glucose oxidase (GOD). Liu et al. proposed an electrochemical method based on the electrochemical–chemical–chemical (ECC) redox cycle with ferrocene methanol (FcM) as the redox mediator and ALP amplification for miRNAs ultrasensitive detection [45]. Lin et al. used the interface engineering strategy of DNA nanostructures to form DNA tetrahedral structures to improve detection specificity. Meanwhile, enzyme amplification technology was used to enhance the output signal and overcome the problems of short size and low abundance in the detection of miRNAs [46]. Guo et al. constructed an electrochemical biosensor platform based on the enzyme catalyzed deposition of DNA conducting molecules (polyaniline) and DNA framework electrochemical background signal conversion strategy (etFNA) for ultra-sensitive and specific detection of miRNAs [47]. In the presence of target miRNAs, tFNA probes allow miRNAs and their partially complementary botanized DNA to assemble a sandwich structure through base stacking effects. Then, in the presence of aniline and H_2_O_2_, HRP catalyzed PANI deposition. TFNA effectively converts the “background” capture probe into a detectable electrical output by generating an electroactive PANI/DNA complex. This signal amplification strategy combines the signal enhancement of enzyme catalysis with the advantages of 3D tFNA in interface regulation and noise signal conversion to achieve ultra-sensitive and specific detection of miRNAs.

Due to the disadvantages of most natural enzymes, it is easy for their catalytic structure to change under harsh environments, such as high heat, strong acids, and strong alkalis, resulting in a reduction in catalytic activity. Therefore, based on the catalytic principle of enzymes, scientists simulated the biocatalytic function of enzymes and synthesized artificially simulated enzymes with high stability and specific catalytic function. Commonly used artificially simulated enzymes include nanozymes, cyclodextrins, high-weight molecular polymers, etc. With the rapid development of nanotechnology, scientists have synthesized nanozymes that not only have the unique physical and chemical properties of nanomaterials but also have enzyme catalytic activity. Nanozymes have a high surface area and good optical and electrical properties, so they are widely used in biosensing technology [48,49]. Compared with natural enzymes and traditional artificial enzymes, nanozymes are simple to prepare, easy to modify, and resistant to acids and bases. In addition, the catalytic activity of nanozymes can be effectively regulated by changing the size, morphology, and surface modification of nanozymes. Ding et al. prepared an electrochemical biosensor based on cascade primer exchange reaction (PER) and MOF@Pt@MOF nanozyme for the detection of exosomal miRNA-21 [50]. In the presence of the target miRNA-21, PER can be triggered to generate long DNA single strands in an isothermal manner. Exposure of the capture probe by the long DNA strand leads to the binding of the nanozyme to the sensing interface. Since the nanozyme is composed of platinum nanoparticles wrapped between two layers of MOF, it shows extraordinary peroxidase catalytic performance, which can decompose H_2_O_2_ to produce significantly amplified electrochemical signals and achieve ultrasensitive detection of exosomal miRNA-21. Su et al. constructed an electrochemical platform based on bimetallic nanozyme and tiptoe-mediated DNA replacement strategy for the sensitive detection of miRNA-21 [51]. AuPt nanoparticles (AuPt NPs) show good peroxidase properties and can catalyze H_2_O_2_ to produce measurable electrochemical signals. To further enhance the detection signal, a new pedicle point-mediated DNA replacement strategy was used. DNA strands with specific sequences were modified on the electrode surface and the nano-enzyme, respectively. In the presence of miRNA-21, continuous cyclic chain replacement can be carried out, resulting in a large accumulation of AuPt NPs on the electrode surface. Subsequently catalyzing H_2_O_2_, a significantly enhanced electrical signal was obtained with a detection limit as low as 84.1 fM.

#### 2.2.2. Nanomaterial-Based Signal Amplification

**Figure 3:**
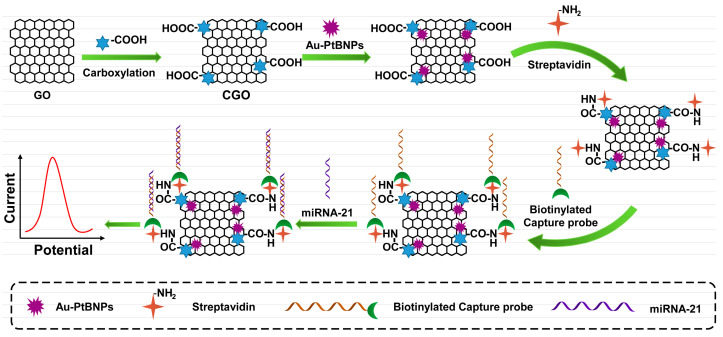
Schematic illustration of based on the CGO decorated with Au–PtBNPs, Specific binding of streptavidin and biotin to capture probe for electrochemical detection.

Nanomaterials refer to materials with at least one nanometer size in the range of 1–100 nm in three-dimensional space that exhibit good electrochemical properties due to their physical properties. Scientists take advantage of the advantages of nanomaterials, such as their good electrical conductivity, large specific surface area, and strong catalytic ability, and apply them to improve the performance of electrochemical sensors and achieve the purpose of improving sensor sensitivity. Au nanoparticles (Au NPs), Pt nanoparticles (Pt NPs), grapheme (GO), quantum dots (QDs), or metal–organic frameworks (MOFs) are commonly used nanomaterials for signal amplification in electrochemical sensors. As shown in Figure 3, Bharti et al. based on the high specific surface area of graphene oxide (GO) and the ability to provide functional groups, such as hydroxyl and carboxyl groups, as well as the good conductivity of gold–platinum bimetallic nanoparticles, prepared an electrochemical biosensor for detecting miRNA-21 [52]. First, the gold–platinum bimetallic nanoparticles were modified on the carboxylated graphene oxide (CGO)-coated fluorine tin oxide (FTO) film, and then the capture probe was immobilized on the modified film through biotin–avidin interaction. Finally, the specificity of miRNA-21 was detected. Dong et al. proposed a proportional electrochemical sensing strategy based on catalytic hairpin assembly target recovery, triggering dual signal output for the ultra-sensitive detection of miRNA [53]. To achieve a ratio dual signaling strategy, an electrochemical indicator, methylene blue (MB), was inserted into the pores of graphene aerogel (GA), and metal–organic framework (MOF) composites and FeMOFs with distinct redox potential separation were selected as the other electrical signal probes. In the presence of target miRNAs, the CHA process can be triggered and the signal probe is introduced to the electrode surface, generating abundant double-stranded H1-H2@FeMOFs-NH_2_. Finally, differential pulse voltammetry (DPV) was used for measurement and analysis. The results showed that the peak current ratio of I_Fe–MOFs_/I_MB_ could accurately reflect the concentration of miRNA.

**Figure 4:**
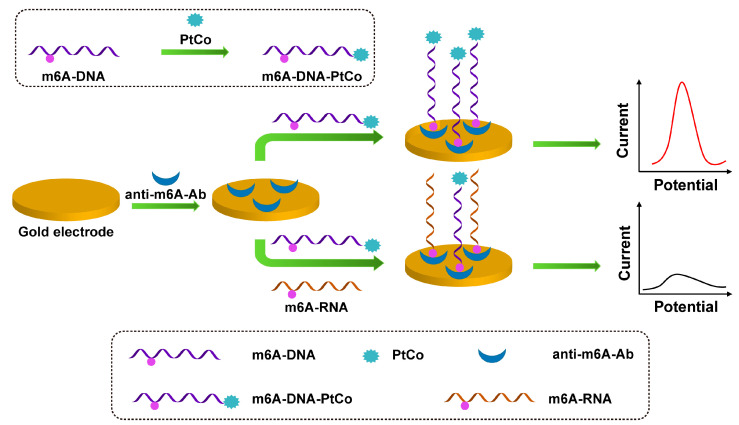
Schematic illustration of the preparation of m6A-DNA-PtCo and the process of m6A-RNA competing with m6A-DNA-PtCo to bind antibodies.

As shown in Figure 4, Xie et al. reported a highly sensitive electrochemical immunosensor for m6A detection based on anti-m6A-Ab and PtCo mesoporous nanospheres (MPN) [54]. Since the anti-m6A antibody can recognize m6A-RNA and m6A-DNA, the m6A-DNA is combined with PtCo MPN to form an M6A-DNA-PtCo probe, which can be used as a signal probe to compete with the target m6A-RNA for binding to the antibody on the electrode surface. PtCo MPN can catalyze H_2_O_2_ to generate electrochemical signals and finally perform DPV scanning to achieve highly sensitive detection of m6A.

#### 2.2.3. Nucleic Acid-Based Signal Amplification

Nucleic acid signal amplification technology has become one of the most effective signal amplification strategies because of its advantages of simplicity, low cost, and high sensitivity. By combining nucleic acid amplification technology with biosensing technology, scientists have constructed various biosensors with high sensitivity and selectivity. The nucleic acid signal amplification methods commonly used in electrochemical biosensors include hybridization chain reaction (HCR), rolling circle amplification (RCA), catalytic hairpin assembly (CHA), etc.

HCR is a nucleic acid amplification technique performed at a constant temperature without enzymes [55]. In the HCR system, the target DNA or RNA triggers two kinds of hairpin DNA continuously, followed by a series of nano-cascade self-assembly amplifications to form long double-stranded DNA (DsDNA) containing a large number of repeating units. HCR has been widely used in the design of electrochemical biosensing due to its advantages of simple operation, high assembly specificity, constant temperature reaction, and no enzyme involvement. Zhao et al. reported an electrochemical biosensor based on tetrahedral DNA nanostructured (TDN) probes, HCR, and horseradish peroxidase catalysis (HPEC) [56]. First, Au NPs were deposited on the surface of the gold electrode to improve the electrical activity and immobilization of the capture probe. The TDN probe was used as the backbone to improve the reactivity. The HCR response is then triggered in the presence of miRNA-122. Finally, through the specific combination of biotin and streptavidin, a large amount of HRP was modified on the surface of the electrode, and HRP catalyzed H_2_O_2_ to generate an amplified electrical signal with a lower limit detection of 0.74 aM.

RCA is a signal amplification method for nucleic acid amplification under isothermal conditions [57,58]. The RCA reaction uses short DNA or RNA strands as primers to hybridize with the designed circular template, and induces the amplification of the primer strands in the presence of polymerase and dNTPs to generate long single-stranded DNA with multiple sets of repeating sequences. The long-chain DNA produced by RCA can be combined with more signal probes to multiply the detection signal. Wang et al. developed a nucleic acid lock (NAL) nanostructure and combined NAL with RCA technology to construct an electrochemical biosensor for highly sensitive detection of miRNA-21 [59]. First, the DNAzyme chain is used as the NAL structure, which is very stable, and miRNA-21 can specifically open the NAL structure. As the structure of NAL changes, it not only releases trigger and target RNA to realize target recycling, but also triggers various signal amplification processes. Then, the cleavage activity of DNAzyme was activated with the assistance of metal ions, and finally the quantitative detection of miRNA-21 was realized by using silver nanoclusters (AgNCs) to catalyze H_2_O_2_ to generate electrochemical signals. This sensing strategy not only has high specificity but also greatly improves signal amplification efficiency and ultrasensitive detection of targets.

CHA is also an isothermal enzyme-free signal amplification method that has attracted widespread attention due to its good enzyme-free signal amplification properties [60,61]. For the CHA amplification reaction, a pair of DNA hairpin probes was first designed, which have good stability and do not react with each other. When the target exists, the hairpin probe can be induced to open the hairpin structure, hybridize to form a stable double-stranded DNA structure, and at the same time displace the target for the next cycle. Zhang et al. prepared a Pd@metal-organic framework (Pd@UiO-66) combined with targeted catalytic hairpin assembly (CHA) to construct an electrochemical biosensor for the highly sensitive detection of miRNA-21 [62]. The proposed biosensing platform not only has an efficient CHA strategy, but also the modified Pd@UIO-66 nanocomposite has excellent electrocatalytic performance, so the sensing technology has a significant amplification effect on the output electrochemical signal. Pd@UiO-66 was used as an electrical signal probe in the sensor platform, and the number of adsorbed probes affected the intensity of the output signal. The results showed that the response current was positively correlated with the content of miRNA-21, which provided a basis for the quantitative detection of miRNA-21. Under the optimal experimental conditions, the detection limit of the sensor was 0.713 fM. The analytical figure of merit for electrochemical immunosensors is shown in Table 1.

## 3. Photoelectrochemical Biosensor

Photoelectrochemistry is a photoelectric conversion phenomenon in which excited-state photomaterials undergo electron excitation and charge transfer under the irradiation of light, resulting in a change in photocurrent. PEC biosensors are constructed based on the principle of photoelectrochemistry. Compared with traditional electrochemical analysis methods, PEC analysis technology shows lower background signal and higher sensitivity [63,64,65]. In addition, PEC biosensors do not require complex instruments and equipment, are low in cost, are easy to miniaturize, and can be used for the highly sensitive detection of life-related substances such as proteins, nucleic acid molecules, cells, small molecules, and ions [66,67,68,69]. Signal amplification strategies for PEC biosensors have been developed, which can be divided into enzyme-catalyzed-based signal amplification, nanomaterial-enhanced signal amplification, and nucleic acid-based signal amplification.

### 3.1. Photoelectrochemical Sensing Analysis

PEC biosensor is composed of a light source, object recognition unit, photoelectric active material, signal converter, and signal output device. In PEC biosensors, an appropriate light source is used to excite photoelectric active electrode materials. The electrode materials absorb photons under light irradiation to produce electron excitation and charge transfer, and redox reactions occur between the electrode materials and electrolyte to generate corresponding photocurrent or photovoltage signals, which finally realize the conversion from light energy to electrical energy. The detection principle of the PEC biosensor is as follows, the sensor can stimulate the photoelectric performance of the corresponding photoelectric material when it is illuminated, that is, generate a photocurrent signal. When the target analyte is captured, this can directly or indirectly change the properties of the photoactive material or electrolyte environment, resulting in a corresponding change in the photoelectric signal. Within a certain range, there is a certain functional relationship between the change value of the photoelectric signal and the concentration of the target substance, and the quantitative detection of the target substance can be realized through this functional relationship.

### 3.2. Signal Amplification Strategies Used in PEC Sensing

Although a variety of PEC biosensors with excellent performance have appeared, the photoelectric properties of optoelectronic materials and sensor design strategies still have significant innovation value. Signal amplification technology is one of the favorable means to improve detection sensitivity in the case of low-abundance targets. To achieve signal amplification, various signal amplification strategies have been developed to achieve highly sensitive bioanalysis of PEC biosensors.

#### 3.2.1. Enzyme-Catalyze Signal Amplification

Enzymes have high catalytic efficiency and substrate specificity, so they are often used for signal amplification of PEC biosensors, such as HRP, GOD, ALP, etc. [70,71,72,73]. Huang et al. developed a cathode-anode space-separated PEC sensing platform based on a dual-electrode synergistic amplification strategy for ultrasensitive detection of miRNA-21 [74]. With good hydrophilicity and biocompatibility, AgInS_2_ nanoparticles can be used as a photoanode to construct the biorecognition step and amplify the signal, while PbS quantum dots are used as a photocathode to enhance the signal. With the participation of ALP, ascorbic acid-2-phosphate is catalyzed to generate ascorbic acid, and ascorbic acid acts as an electron donor, which enhances the PEC signal and improves the detection sensitivity.

With the rapid development of biomimetic materials and nanotechnology, many artificial enzymes with excellent properties have been developed, including porphyrins, metal oxides, and hemin [75,76]. Li et al. reported a cascaded amplification PEC sensing strategy for highly sensitive detection of miRNA-21 [77]. In this sensing strategy, the DNA probe is combined with the target to generate G-quadruplex (G4) forming sequences through an enzyme cleavage reaction, and the released target at the same time initiates the subsequent cycle process, which can generate a large number of G4 forming sequences. The G4 sequence combines with hemin to form G4/hemin DNAzyme, which has a catalytic activity similar to that of peroxidase, and can catalyze the production of 1, 4-benzenediol (BQ) for conjugating onto the surface of the chitosan (CS) deposited BiOI/ITO photocathode. Under light excitation, BQ can act as the electron acceptor of BiOI to promote the generation of photocurrent, so as to achieve the purpose of detecting target RNA with high sensitivity and specificity. Wang et al. prepared a novel AuPt nanodendrites (NDs) as a nanoenzyme and constructed a PEC biosensing platform for the sensitive detection of miRNA-141 based on a sensitive heterogeneous structure BiVO_4_/CoPi [78]. The AuPt NDs-labeled DNA probe (pDNA) can be bound to the electrode surface in the presence of the target RNA. The AuPt NDs nanoenzyme can effectively catalyze the oxidation of 3-amino-9-ethylcarbazole (AEC) in the presence of H_2_O_2_ to form a precipitate on the photoanode. The detection results showed that the addition of AuPt NDs labeled DNA probe (pDNA) resulted in a sharp decay of the photocurrent on the developed sensor, which eventually led to the sensitive detection of miRNA-141.

#### 3.2.2. Nanomaterial-Enhanced Signal Amplification

**Figure 5:**
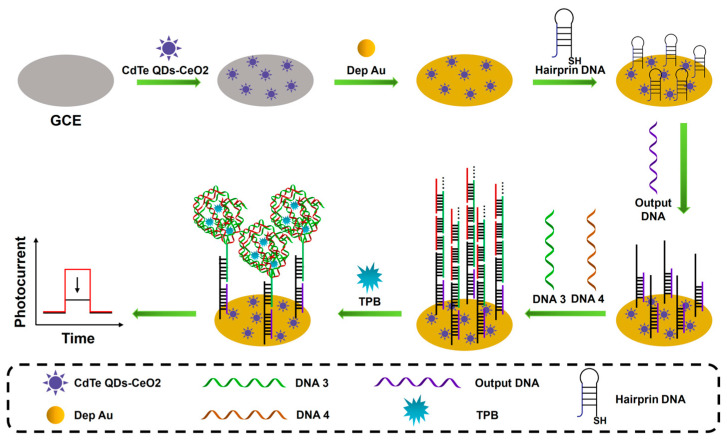
Schematic illustration of the CdTe QDs-CeO_2_ complex as the PEC signal indicator; with the addition of TPB, the generated DNA super-sandwich structure would form DNA-protein complexes, leading to a significantly reduced PEC signal. (The red line represents the original sensing signal, the black line represents the sensing signal based on signal amplification. This is a negatively correlated signal amplification).

The nanomaterial-enhanced signal amplification is more and more widely used in PEC sensing analysis due to its unique electrical and optical advantages. In order to further improve the photoelectric conversion efficiency of photoactive materials and increase the immobilization of biomolecules, various types of photoelectric composite materials have been developed, such as semiconductor–metal composites and semiconductor–carbon composites. As shown in Figure 5, Li et al. proposed a PEC biosensor based on CdTe quantum dot-CeO_2_ complexes (CdTe QDs-CeO_2_ complex) and a DNA super-sandwich structure dual signal amplification strategy for ultrasensitive detection of miRNA-141 [79]. The CdTe QDs-CeO_2_ complex can reduce energy loss and significantly improve the photoelectric conversion efficiency of the sensor. In addition, a targeted conversion amplification program based on DNA Walker is also used to achieve the purpose of converting a small amount of target RNA into a large amount of DNA. A DNA–protein complex is formed with the participation of binding proteins, which effectively block electron transport and ultimately lead to a significant weakening of the output PEC signal. Miao et al. constructed a PEC biosensor for highly sensitive detection of miRNA-21 based on a Ti_3_C_2_/MgIn_2_S_4_ heterojunction and a multifunctional porphyrin metal–organic framework (PCN-224) [80]. The Ti_3_C_2_/MgIn_2_S_4_ heterojunction effectively improved the photoelectric conversion efficiency and was able to provide a strong initial photocurrent signal. Meanwhile, the porous PCN-224 serves as a good nanocontainer for the signal amplification molecule glucose using a capture probe (CP) encapsulation. In the presence of miRNA-21, glucose molecules were released from PCN-224 and oxidized in situ by glucose oxidase (Gox) to generate hydrogen peroxide, which cleared the pores of Ti_3_C_2_/MgIn_2_S_4_ and significantly enhanced the photocurrent response. This sensing strategy exhibited good miRNA-21 detection performance with a low detection limit of 0.17 fM.

#### 3.2.3. Nucleic Acid-Based Signal Amplification

Nucleic acid amplification signal amplification technology has become one of the most effective ways to improve the detection of RNA output signals of PEC biosensors due to its good specificity and the advantages of cascade amplification.

**Figure 6:**
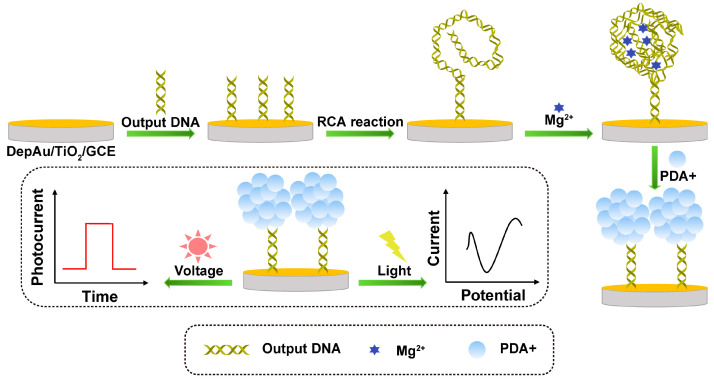
Schematic illustration of the formation process of DNA nanospheres and the PEC and ECL detection of dual-mode biosensors.

RCA is an enzyme-assisted constant temperature nucleic acid amplification technology that can not only realize the amplification of DNA and RNA but also be used to amplify the signal of the target nucleic acid to generate long-chain DNA with multiple sets of repeated sequences, so that the PEC sensor has extremely high detection sensitivity. As shown in Figure 6, Deng’s team developed a photoelectrochemical and electrochemical dual-mode biosensor that can obtain extremely strong PEC signals and obvious ECL signals to achieve sensitive and accurate detection of miRNA-141 [81]. First, a large amount of DNA is generated by cleavage cycling of the ternary Y structure associated with the target, and the DNA is immobilized on a TiO_2_ substrate, thereby initiating the RCA reaction. Then, with the assistance of magnesium ions and PDA^+^, the RCA products formed multifunctional DNA nanospheres. Due to the short distance between the DNA nanospheres and the TiO_2_ substrate, a PDA^+^-TiO_2_ sensitized structure was formed, which was able to generate a strong PEC signal with the aid of ascorbic acid (AA). At the same time, PDA^+^ has good redox activity, so it can also produce clear cathode peaks and obtain obvious ECL signals.

HCR is driven by a strand displacement reaction in an isothermal and enzyme-free environment, and a primer strand triggers two hairpin DNAs to continuously hybridize with each other, eventually forming a long double-stranded DNA. Zhao’s group proposed a novel PEC biosensor for ultrasensitive detection of miRNA-21 [82]. The sensing platform exhibits excellent sensing performance based on an enzyme-free nucleic acid double amplification strategy and a simulated enzyme-catalyzed precipitation quencher. Entropy-driven strand displacement reaction (ESDR) amplification and HCR amplification were performed first, and the synergistic effect of the dual amplification strategy resulted in the formation of long double-stranded DNA triggered by miRNA-21. Manganese porphyrin (MnPP) is a substance similar to HRP, which embeds MnPP in long DNA double strands and catalyzes the conversion of benzo-4-chlorohexadienone on the electrode surface, resulting in a significant decrease in the PEC signal and showing highly sensitive detection of miRNA-21. Wang et al. constructed a PEC biosensing platform for miRNA-375-3p ultrasensitive detection based on a novel photoactive poly (3, 4-ethyl-enedioxythiophene) (PEDOT)/FeOOH/BiVO_4_ nanohybrid and an enzyme-free signal amplification strategy [83]. Compared with the conventional FeOOH/BiVO_4_ photoactive composite, PEDOT/FeOOH/BiVO_4_ has better photoelectric conversion efficiency and exhibits significant photocurrent enhancement. Using PEDOT/FeOOH/BiVO_4_ as the photoelectrode and enzyme-free signal amplification strategies, such as target-induced catalytic hairpin assembly (CHA) and hybridization chain reaction (HCR), the response signal was greatly improved to achieve sensitive detection of miRNA-375-3p with a detection limit as low as 0.3 fM.

CHA is a DNA-hairpin self-assembly technology without the participation of enzymes. Because of its advantages of simple and flexible operation, high sensitivity, and strong specificity, it is a commonly used nucleic acid signal amplification strategy in the field of PEC biosensing. Zhao et al. reported a synergistic strategy based on nucleic acid signal amplification, enzyme-catalyzed amplification, and chemical signal amplification for ultrasensitive detection of miRNA-21 [84]. In this PEC biosensor, CdS NPs-modified carbon cloth (CC) on polyimide (PI) film is employed as a photoelectric material to become a photoelectrode. In the presence of the target miRNA-21, the nucleic acid signal amplification reactions (CHA and HCR) are triggered, producing long dsDNA labeled with biotin. Then, the enzyme-catalyzed reaction is carried out, and ALP captured by the DNA strand catalyzes the production of ascorbic acid (AA), which finally generates a significant photoelectric signal, realizing the ultra-sensitive detection of miRNA. The analytical figure of merit for photoelectrochemical immunosensors is shown in Table 2.

## 4. Fluorescent Biosensor

A fluorescence biosensor is a kind of optical biosensor that uses biomolecules as recognition elements, uses fluorescence signal changes caused by fluorescence resonance energy transfer or electron transfer as detection signals, and finally realizes qualitative and quantitative analysis of biological samples. Compared with traditional biosensors, fluorescent biosensors not only have the characteristics of strong selectivity but also combine the advantages of fluorescence analysis technology, such as simple equipment, low cost, fast response, and high analytical sensitivity, and have important application value in many fields, such as clinical disease diagnosis, environmental monitoring, and food testing [85]. In order to improve the recognition and transduction processes of fluorescent biosensors and then improve the scope and detection sensitivity of fluorescence detection, it is necessary to introduce appropriate fluorescence signal amplification strategies. Nucleic acid is the most commonly used signal amplification carrier in fluorescent biosensors. It has strong specific recognition ability and cascade amplification ability.

### 4.1. Fluorescent Sensing Analysis

Fluorescence groups absorb photons of a certain energy after being excited by external light energy so that the electrons in the ground state (S0) absorb energy and transition to the excited state (S1 or S2). However, electrons in the excited state are unstable and will soon release energy back to the first electron-excited singlet state (S1) by means of non-radiative transition and internal transformation. The light emitted by some electrons in the S1 level returning to the ground state (S0) by means of radiation transition is fluorescence. The basic principle of fluorescent biosensors is to convert the recognition signal into an optical signal expressed by the photophysical properties of the fluorophore by capturing the target molecule, and then use the enhancement or weakening of the fluorescence signal to realize the detection of the target. Many materials that emit fluorescent signals have been found, including organic fluorescent materials, semiconductor quantum dots, graphene quantum dots, carbon quantum dots, etc. These fluorescent materials are often widely used as fluorescent probes in fluorescent biosensing.

### 4.2. Signal Amplification Strategies Used in Fluorescent Sensing

There are usually two ways to improve the sensitivity of fluorescent biosensing platforms, one is to reduce the background signal of the reaction system, and the other is to use nucleic acid molecular signal amplification technology to increase the signal output of the reaction system. Since nucleic acid molecular signal amplification technology can significantly improve the sensitivity of fluorescent biosensors and has high specificity, it has become a research hotspot in the field of fluorescent biosensing. According to whether there are tool enzymes involved in the signal amplification process, nucleic acid signal amplification can be divided into two types, enzyme-assisted and enzyme-free.

#### 4.2.1. Enzyme-Based Nucleic Acid Signal Amplification

In enzyme-assisted nucleic acid signal amplification technology, commonly used enzymes include DNA polymerase, endonuclease, and exonuclease. Currently commonly used amplification techniques include polymerase chain reaction (PCR) [86], strand displacement amplification (SDA) [87], the RCA reaction [88,89,90], etc.

SDA is an enzymatic DNA amplification technique under isothermal conditions that has the advantages of simple operation and high amplification efficiency. In the SDA reaction, under the action of restriction endonuclease and DNA polymerase, the target molecule and the primer can combine with the primer and be continuously extended and replaced, thereby generating a large number of target complementary strands and achieving efficient amplification of the target sequence [91,92]. Guo’s group reported a fluorescent biosensor based on a self-sufficient fuel amplification strategy (SFAS) to achieve label-free and highly sensitive detection of miRNA-21 [93]. This sensing strategy first connects two SDA processes in a series for cascaded amplification of single-stranded DNA. Single-stranded DNA can partially hybridize with the hairpin template and elongate along the template under the action of DNA polymerase, resulting in the destruction of the hairpin structure. AgNCs are modified on the hairpin template, and the fluorescence of AgNCs depends on the wrapping of the hairpin structure, so the destruction of the hairpin structure leads to the quenching of the fluorescence of AgNCs, and, finally, the fluorescence of miRNA-21 is weakened.

**Figure 7:**
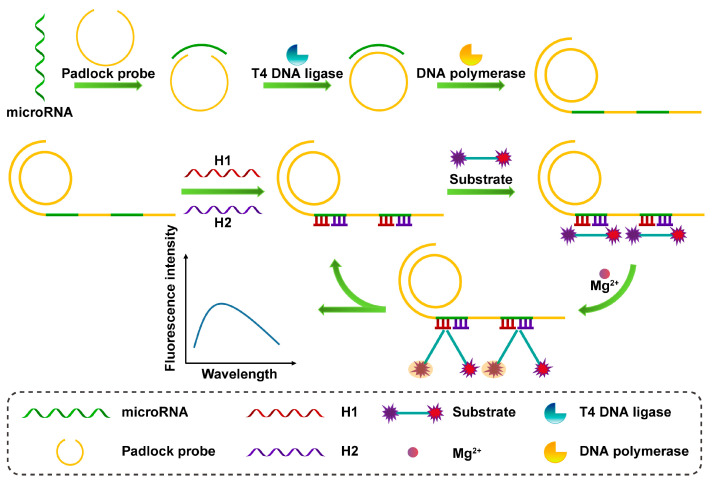
Schematic illustration of the RCA process and the fluorescent assay of the DNAzymes cleavage substrates.

The RCA reaction is an isothermal nucleic acid amplification method using a circular template that has high sensitivity and specificity and is often used for signal amplification of fluorescent biosensors. As shown in Figure 7, Fang et al. proposed a fluorescent biosensing strategy based on an RCA system and an allosteric DNAzymes system for highly sensitive detection of miRNA-21 [94]. The target-driven RCA reaction generates long-chain DNA with many repeating units, and after adding single-stranded DNA (H1 and H2), hybridization forms a multicomponent nuclease (MNAzyme) with the ability to cleave the substrate. Finally, substrates containing fluorescent groups and quenching groups and magnesium ions are added to the system, and the MNAzyme is activated by the magnesium ions to cut the substrate to enhance the fluorescence intensity. Lee et al. proposed a fluorescence resonance energy transfer (FRET) strategy for the highly sensitive detection of miRNA-21 based on PstI endonuclease cleavage (PEC) and RCA [95]. The miRNA-21 was able to specifically initiate the RCA reaction and the synthesized RCA products self-assembled to form long hairpin DNA (HD) structures. In the presence of the PstI enzyme, the structure can be specifically recognized and cleaved by the PstI endonuclease. This will release a large number of short single-stranded DNA fragments that complementarily hybridize with HD probes labeled with FAM on one end and BHQ-1 on the other end and open the stem-loop, resulting in reduced FRET efficiency. The detection limit of miRNA-21 was 39.7 aM.

#### 4.2.2. Enzyme-Free Nucleic Acid Signal Amplification

Enzyme-assisted nucleic acid signal amplification technology requires the participation of enzymes, but the reaction system may affect the activity of enzymes. Enzyme-free nucleic acid signal amplification has the advantage of simple operation and can be used to develop novel fluorescent biosensors with a simple design and high sensitivity. Commonly used non-enzyme-assisted signal amplification strategies include the HCR reaction [96,97,98], the CHA reaction [99,100], etc.

HCR signal amplification can maintain high specificity for target molecules without enzyme assistance, and the operation process is relatively simple, so it has great application value in fluorescent biosensing. Dong’s team proposed a fluorescent biosensing platform based on the combination of a constant temperature network HCR and reverse transcription-rolling circle amplification (RT-RCA) for the detection of circRNA [101]. First, a specific RT-RCA reaction is performed on the target circRNA. The target circRNA can be reverse-transcribed into a single-stranded cDNA with multiple back-splicing junctions, which can be used as a target for subsequent HCR reactions, while linear RNA can only produce one reaction. Then, three probes were designed for the HCR reaction. The existence of the back-splicing adapter can change the conformation of the trigger probe, thereby triggering the continuous strand replacement process between the two hairpin probes H1 and H2, and, finally, forming a stable DNA nano-network structure, which greatly enhances the fluorescence signal of the system. Zada et al. proposed a simple miRNAs detection and imaging strategy based on Mo_2_B nanosheets (NSs) and hybrid chain reaction (HCR) [102]. Mo_2_B NSs not only provide large surface area loading for hairpin probes (HPs) and ssDNA loops, but also have excellent multifluorescent dye quenching performance for ultra-low background signal. After transfection, HPs recognize specific target miRNAs and trigger HCR to produce a large number of DNA-miRNA double helices, which dissociate from the surface of Mo_2_B NSs and generate strong fluorescent signals for miRNAs detection. Finally, the detection and imaging of miRNAs in different cells were achieved.

**Figure 8:**
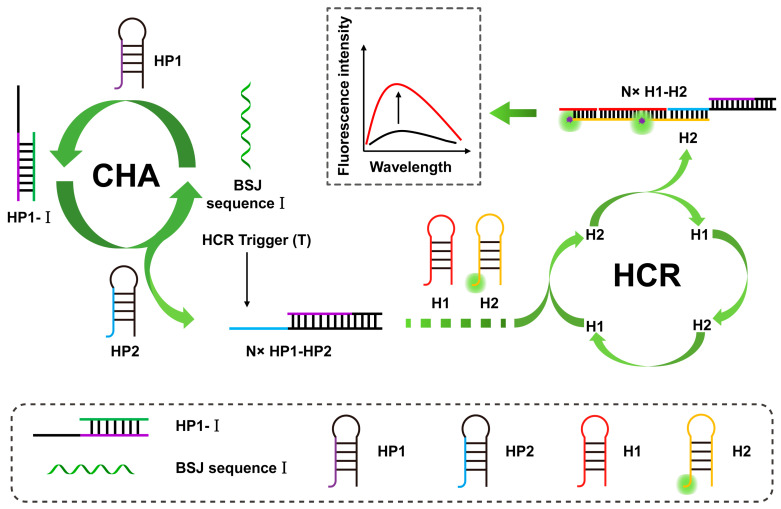
Schematic illustration of BSJ sequence I triggering the CHA–HCR cascade amplification system. (The black line represents the original sensing signal, the red line represents the sensing signal based on signal amplification. This is a positively correlated signal amplification).

The triggering process of the CHA reaction is similar to that of the HCR reaction, except that the trigger probe can be released to participate in the next cycle, while in the HCR reaction, the trigger chain will not be released after being combined with the hairpin probe. As shown in Figure 8, Li et al. developed a highly selective detection system (GO-CHA-HCR) based on the combination of CHA, HCR, and graphene oxide (GO) for the imaging of CIRC-Foxo3 and circRNA in living cells [103]. The system includes four specially designed hairpin probes, one of which is labeled with a fluorescent group. These are adsorbed on the surface of GO nanosheets, and GO acts as a quencher to quench the fluorescence. Next, in the presence of the target, the CHA cycle is initiated to form a hybrid strand, which triggers the HCR cycle to generate long dsDNA, which is then released. The quenched fluorescence in this process is recovered, realizing double signal amplification in the detection process. The analytical figure of merit for fluorescent immunosensors is shown in Table 3.

Electrochemical, photoelectrochemical, and fluorescent biosensing technologies have brought many benefits to all aspects of human life, especially providing more possibilities for early diagnosis of cancer. These three biosensors have many advantages, such as being simple and easy to obtain, low cost, fast response time, high detection sensitivity, etc. However, they also have disadvantages, such as low stability, ease to be interfered with, and easy fluorescence quenching. The advantages and disadvantages of electrochemical, photoelectrochemical, and fluorescent biosensors are shown in Table 4.

## 5. Conclusions and Future Outlook

This review highlights the important role of RNA tumor markers in cancer. Rapid and highly sensitive detection of RNA tumor markers by biosensors is used for early diagnosis, prognosis, and treatment of malignancies. Biosensors have been shown to be a simple, accurate, and rapid sensing technology for the detection of cancer tumor markers. Due to the low abundance of RNA tumor markers in blood and the complex detection environment, highly sensitive and excellently specific detection of RNA tumor markers requires signal amplification strategies to enhance the detection signal or reduce the background signal. In this paper, three kinds of biosensors with different signal transduction modes are reviewed, including the electrochemical biosensor, the photoelectrochemical biosensor, and the fluorescent biosensor. Signal amplification strategies applied to sensors include enzyme-catalyzed, nanomaterial-based, and nucleic acid-based strategies of signal amplification. In the current study, many biosensing platforms have been successfully applied to the detection of samples from clinical cancer patients. Therefore, we are confident that in the near future, biosensors will have broader prospects in clinical diagnosis and prognosis monitoring. However, the effects of RNA tumor markers on the progression of cancer cells need to be continuously investigated. Similarly to the mechanisms of interaction between the signal amplification strategies of individual biosensors and RNA tumor markers, both need to be continuously explored and investigated. It is expected that more responsive, sensitive, and selective biosensing technologies will be discovered for cancer research in the future.

## Figures and Tables

**Table 1:** Analytical performance characteristics of reported electrochemical immunosensors.

Immunosensor Components	Target Analyte	Detection Method	Linear Range	Detection Limit	Ref.
miRNA-29b-1/p-29b-1/CP/RGO/GCE.	miRNA-29b-1	SWV	1 fM–1 nM	5 fM	[43]
RNase A/m6A-RNA/ anti-m6A-Ab/Au	m6A-RNA	EIS	0.05 nM–200 nM	0.016 nM	[44]
miRNA-21/SA-ALP/Au	miRNA-21	EC	0.5 fM–1 pM	0.2 fM	[45]
biotin-DNA-miRNA-21/tFNA/Au	miRNA-21	DPV	1 fM–1 nM	290 aM	[47]
MOF@Pt@MOF nanozyme/GCE	miRNA-21	DPV	1 fM–1 nM.	0.29 fM	[50]
miRNA-21/DNA@AuPt/Au	miRNA-21	i-t	0.1 pM–1 nM	84.1 fM	[51]
miRNA-21/CP/SA/Au-PtBNPs/CGO/FTO	miRNA-21	DPV	1 fM–1 μM	1 fM	[52]
H1-H2@Fe-MOFs-NH_2_/miRNA-155/AuNPs/MB-GA-UiO-66-NH_2_/GCE	miRNA-155	DPV	1 fM–100 nM	50 aM	[53]
m6A-RNA/m6A-DNA-PtCo/anti-m6A-Ab/Au	m6A-RNA	DPV	0.005 nM–100 nM	2.1 pM	[54]
TDN/miR-122/HPEC/AuNP/Au	miRNA-122	EC	1 aM–100 fM	0.74 aM	[56]
RCA/AgHPs/miRNA-21/NAL/Au	miRNA-21	DPV	10 aM–100 pM	9.3 aM	[59]
H2/Pd@UiO-66/CHA/miRNA-21/H1/GE	miRNA-21	DPV	20 fM–600 pM	0.713 fM	[62]

SWV: Square wave voltammetry, EIS: Electrochemical impedance spectroscopy, EC: Electrochemistry, DPV: Differential pulse voltammetry, i-t: Current-time curve.

**Table 2:** Analytical performance characteristics of reported electrochemiluminescent immunosensors.

Immunosensor Components	Target Analyte	Detection Method	Linear Range	Detection Limit	Ref.
NP–DNA/ALP-Au/AgInS_2_/PbS	miRNA-21	PEC	10 fM–100 nM	3.4 fM	[74]
G4/hemin/CS/BiOI/ITO	miRNA-21	PEC	1.0 fM–0.1 nM	0.2 fM	[77]
H_2_O_2_-AEC/pDNA-AuPt NDs/miRNA-141/cDNA/CoPi/BiVO_4_	miRNA-141	PEC	5 fM–1 pM	0.17 fM	[78]
TBP/TATA/HT/DNA/depAu/CdTe QDs-CeO_2_/GCE	miRNA-141	PEC	0.5 fM–5 nM	0.17 fM	[79]
miRNA-21/CP/PCN-224/Ti_3_C_2_/MgIn_2_S_4_	miRNA-21	PEC	0.5 fM–1.0 nM.	0.17 fM	[80]
DNA/HT/outpour DNA/depAu/TiO_2_/GCE	miRNA-141	PEC	0.1 fM–1 nM	0.037 fM	[81]
		EC	2 fM–500 pM	0.67 fM	
H_2_O_2_+4-CN/MnPP/output DNA/HT/H1/DepAu/BiOCl-BiOI/GCE	miRNA-21	PEC	100 aM–1 nM	33 aM	[82]
MB/HP3-HP4/miRNA-375-3p/PolyA-HP1/PEDOT/FeOOH/BiVO_4_	miRNA-375-3p	PEC	1 fM–10 pM	0.3 fM	[83]
ALP-SA/HP4-Biotin/HP3/HP2/miRNA-21/HP1/CdS/CC	miRNA-21	PEC	1 fM–1 nM	0.41 fM	[84]

PEC: Photoelectrochemistry, EC: Electrochemistry.

**Table 3:** Analytical performance characteristics of reported fluorescent immunosensors.

Immunosensor Components	Target Analyte	Detection Method	Linear Range	Detection Limit	Ref.
Ag NCs/SFAS/miRNA-21	miRNA-21	FL	0.02 nM–1 nM	0.178 pM	[93]
MgCl_2_/MNAzymes/RCA/miRNA-21	miRNA-21	FL	10 pM–50 pM	4 pM	[94]
Hairpin DNA/RCA/miRNA-21/circular dumbbell DNA	miRNA-21	FL	1 aM–1 pM	39.7 aM	[95]
HCR/RT-RCA/circRNA	circRNA	FL	0.1 pM–150 pM	0.1 pM	[101]
DNA-miRNA/HCR/miRNA-21/HPs/Mo_2_B	miRNA-121	FL	1 fM–1 nM,	20.2 fM	[102]
GO-CHA-HCR	circRNA	FL	30 pM–60 nM	10 pM	[103]

FL: Fluorescence.

**Table 4:** The advantages and disadvantages of electrochemical, photoelectrochemical, and fluorescent biosensors.

Biosensors	Advantages	Disadvantages
Electrochemical	Simple equipment, low cost, fast response time, high sensitivity, good selectivity	Storage time is short, stability is not good enough, the detection is easily interfered by other substances
Photoelectrochemical	Low background signal, simple and fast, high sensitivity, good accuracy, high stability	Light may damage biomolecules, while the absorption or scattering of light by biomolecules may interfere with the detection signal, and there is also a low detection throughput
Fluorescent	Low cost, easy to modify, fast response time, high sensitivity, good selectivity	With many interfering factors, fluorescent probes are prone to photolysis, oxidative quenching, and easy contamination.

## Data Availability

Not applicable.
